# Reference Standard for Validation of Age-Related Macular Degeneration Screening Algorithms

**DOI:** 10.1016/j.ophtha.2026.04.013

**Published:** 2026-04-17

**Authors:** Amitha Domalpally, Emily Y. Chew, Malvina B. Eydelman, Tiarnán D.L. Keenan, Pearse A. Keane, Aaron Y. Lee, Cecilia S. Lee, Eleonora M. Lad, Jennifer I. Lim, Anat Lowenstein, Ursula Schmidt-Erfurth, Michael D. Abramoff

**Affiliations:** 1 Wisconsin Reading Center, Department of Ophthalmology and Visual Sciences, University of Wisconsin―Madison, Madison, Wisconsin.; 2 Division of Epidemiology and Clinical Applications, National Eye Institute, National Institutes of Health, Bethesda, Maryland.; 3 Collaborative Community on Ophthalmic Innovation, Reston, Virginia.; 4 NIHR Biomedical Research Centre at Moorfields Eye Hospital NHS Foundation Trust and UCL Institute of Ophthalmology, London, United Kingdom.; 5 Department of Ophthalmology and Visual Science, Washington University in St Louis, Saint Louis, Missouri.; 6 Department of Ophthalmology, Duke University Medical Center, Durham, North Carolina.; 7 Department of Ophthalmology and Visual Sciences, University of Illinois at Chicago, Chicago, Illinois.; 8 Department of Ophthalmology and Optometry, Tel Aviv Medical Center, Tel Aviv, Israel.; 9 Medical University of Vienna, Vienna, Austria.; 10 Department of Ophthalmology and Visual Sciences, Electrical and Computer Engineering, University of Iowa, Iowa City, Iowa.

**Keywords:** Age-related macular degeneration, AI screening, Reference standard, Retinal imaging

## Abstract

**Purpose:**

Artificial intelligence (AI)-based screening models hold promise for identifying individuals with undiagnosed age-related macular degeneration (AMD) in nonspecialist settings. A standardized reference framework for image labeling is needed to enable consistent training, validation, and deployment of AI-based screening algorithms. The goal of the present study was to establish expert consensus on an image-based reference standard for labeling AMD.

**Design:**

Modified Delphi consensus study.

**Participants:**

Fellowship-trained retina specialists, ophthalmologists, AI specialists, and imaging specialists.

**Methods:**

A prespecified Delphi process was conducted using structured surveys. Over 2 rounds, panelists assessed opinions on existing reference standards, including the Age-Related Eye Disease Study scale and Beckman scale, as well as imaging methods such as color, OCT, and autofluorescence. The surveys also evaluated imaging features of AMD, including drusen, pseudodrusen, and pigment changes, as well as referral criteria. Consensus was defined using a 9-point Likert scale, with predefined statistical thresholds for agreement.

**Main Outcome Measures:**

Agreement on key elements of a reference standard.

**Results:**

Consensus was reached on adopting the Beckman classification as the level 1 reference standard (median score, 8; agreement). OCT use for identifying key AMD features, including drusen, geographic atrophy (GA), and choroidal neovascularization, also reached consensus (median scores, 8.5–9; agreement). Pigment change detection did not reach consensus (median, 7.5; uncertain), and screening age thresholds showed nonconsensus (median, 8; uncertain). Referral thresholds reached consensus, including urgent referral for neovascular AMD and nonurgent referral for GA and intermediate AMD (median, 9; agreement).

**Conclusions:**

This study defined a consensus-based reference standard for labeling AMD from images for AI-based screening. These recommendations are intended to support consistent AI model development and evaluation, while remaining distinct from clinical practice guidelines.

**Financial Disclosure(s):**

Proprietary or commercial disclosure may be found in the Footnotes and Disclosures at the end of this article.

Age-related macular degeneration (AMD) is a leading cause of blindness globally, affecting an estimated 18.34 million (11.64%) individuals in the United States with early-stage disease and 1.49 million (0.94%) with late-stage AMD, according to the United States Centers for Disease Control and Prevention.^1^ The disease progresses slowly from early and intermediate stages to advanced disease, defined by geographic atrophy (GA) or neovascular AMD. Although late AMD carries high risk of irreversible vision loss, the earlier stages often are relatively asymptomatic. Consequently, many individuals with early or intermediate AMD remain without a diagnosis until significant vision loss or visual symptoms of distortion prompt them to seek care.^2^ Early identification of these individuals is critical, not only to help patients preserve vision through preventive strategies such as use of Age-Related Eye Disease Study (AREDS) 2 supplements, but also to encourage self-monitoring and lifestyle modification.^3^ Early identification also may facilitate the recruitment of patients into clinical trials to evaluate new treatments for AMD and may ensure timely access to emerging therapies.^4,5^

Screening for AMD is particularly important in the era of emerging therapies for dry AMD, because it facilitates timely intervention and enables self-monitoring with Amsler grid or the ForeSee Home device (Notal Vision Inc) and lifestyle modifications such as smoking cessation, exercise, and healthy dietary habits, specifically the use of the Mediterranean diet, that are known to slow disease progression.^6^ Artificial intelligence (AI)-based screening models hold promise for addressing this gap because they can enable the rapid identification of individuals at risk of AMD in nonspecialist settings and already are improving outcomes for diabetic retinal disease.^7^ However, advancing AI models for AMD screening presents unique challenges, compared with those for other retinal diseases.^8^ These include the need for large multimodal training datasets and consistent labeling across the datasets, which remain far less developed for AMD compared with diabetic retinopathy (DR).^9^

For DR, the United States Food and Drug Administration (FDA) validation pathway is well established by the predicate device using the de novo pathway.^10^ Retrospective data are used to train models, and prospective trials in the intended workflow in primary care clinics are conducted comparing against a human-graded reference standard. In the context of regulatory evaluation of screening algorithms, a reference standard defines how disease presence, severity, and referral urgency are labeled from images for performance benchmarking, rather than how patients are treated in clinical practice. These labels function as ground truth comparators for algorithm evaluation and are not intended to dictate clinical management decisions. Abràmoff et al^11^ have outlined 4 levels of reference standards. Level 1 standards are linked to clinically meaningful visual outcomes and are determined by independent reading centers, and level 2 standards are based on a prespecified, reproducible reading center protocol when outcome linkage is not available. For DR, a level 1 prognostic reference standard (predicting outcome) corresponds to the Early Treatment Diabetic Retinopathy Study grading system and Diabetic Retinopathy Clinical Research Network grading paradigm for center-involved macular edema, both of which have strong visual correlations in large clinical trials.^12–14^ These steps have facilitated regulatory evaluation of AI algorithms for DR screening.

In contrast, for AMD, although several classification systems exist, including the Beckman classification,^15^ AREDS categories,^16^ the AREDS Simplified Severity Scale,^17^ and the 12-step AREDS Severity Scale,^18^ currently no agreement exists regarding a suitable reference standard for regulatory validation of AI-based screening models. Within the scope of this work, the AMD reference standard represents a human-graded, image-based ground truth for screening validation that defines how AMD is categorized from images to support referral decisions by AI models, without extending into treatment or clinical management. Substantial variability in AMD definitions across existing classification systems, as well as differences arising from the imaging method used (e.g., color fundus photography vs. OCT) complicate AI performance benchmarking. Establishing a consistent reference standard at the screening stage therefore is a necessary prerequisite for validation of AI-based screening algorithms.

This study focused on defining the reference standard required for regulatory validation of AI-based screening models for AMD. Using a Delphi process, this work sought to establish a consensus among experts on the optimal standard for validating AI for AMD screening, paving the way for regulatory validation of AI models that can expand AMD care. The consensus aimed to address how AMD should be identified and staged by AI algorithms and how these classifications map to referral categories and timelines for triage in a screening environment outside specialist eye care.

## Methods

This study used the Delphi process to achieve expert consensus on reference standards for the validation of AI-based screening tools for AMD. The Delphi process followed the same method-ology as previous ophthalmology studies and was conducted over 2 rounds, with a supplemental round addressing specific areas of ambiguity.^15,19^ The study was coordinated by the Collaborative Community on Ophthalmic Innovation, an international panel of AMD experts, including ophthalmologists, imaging specialists, and researchers.^20^ The participants were international experts in AMD research, clinical care, and imaging and were involved in the Collaborative Community on Ophthalmic Innovation. Of the 13 invited panelists, 11 participated in round 1. Round 2 also included 11 participants, with 10 individuals participating in both rounds. The supplemental round included 8 participants, all of whom had completed round 2. Experts from the FDA participated in the discussions but were not involved in the consensus voting. (The FDA participates as a member of the Collaborative Community on Ophthalmic Innovation.^20^ The FDA did not contribute to the development of this consensus statement, and the consensus statement should not be construed to represent FDA’s views or policies.)

Anonymity in survey responses was maintained throughout to minimize bias and foster open, uninfluenced contributions. Participation was voluntary, and consent was obtained from all panelists. Institutional review board approval was not required because of the nature of the study. All research adhered to the tenets of the Declaration of Helsinki. A flow summarizing the rounds is available as Figure S1 (available at www.aaojournal.org).

### Delphi Round 1: Qualitative Exploration

The first round of the Delphi process was designed to explore expert opinions on the fundamental aspects of AMD reference standards and to identify areas requiring further discussion. A qualitative survey was designed based on topics and questions submitted by the FDA, which was not part of the voting panel. These topics were reviewed independently by 2 participants (A.D. and E.Y.C.) and were used to structure the round 1 qualitative survey. Participants completed a structured qualitative survey, which included open-ended questions on the applicability of existing grading systems, such as the Beckman classification and AREDS categories, as shown in Supplement 1 (available at www.aaojournal.org). Additional questions examined the role of imaging methods, including OCT, color fundus photography, and fundus autofluorescence, in defining AMD reference standards. Details on imaging protocols were discussed, including scan density for OCT images and number of fields for color photography. The survey also addressed the definitions of AMD stages for screening purposes and the criteria for referral urgency. Responses from this round were analyzed thematically to identify areas of agreement, divergence, and ambiguity, and a summary of the discussion is also included in Supplement 1. These findings were used to create structured statements for evaluation in the subsequent Delphi round by the same 2 participants. No attempt was made to force consensus during round 1 discussions; areas of disagreement were preserved intentionally and were carried forward for structured evaluation in round 2.

### Delphi Round 2: Quantitative Evaluation

The second round focused on achieving consensus through a quantitative evaluation of the responses to the statements derived from round 1, as shown in Table 1. Participants were asked to rate each statement using a 9-point Likert scale, where 1 represented strong disagreement and 9 represented strong agreement. Statements included evaluations of the appropriateness of the Beckman classification as a reference standard, the inclusion of multimodal imaging in reference standards, and the prognostic value of specific features such as reticular pseudodrusen.

All scores were evaluated first for consensus or nonconsensus; scores achieving consensus then were categorized as agree or disagree. To determine the level of consensus, statistical criteria were applied, similar to those used in the development of the Beckman classification.^15^ Consensus was defined as no more than 20% of responses falling outside the 3-point bracket containing the median. Statements were classified as nonconsensus if 33% or more of responses fell into both the polar ranges of 1 through 3 and 7 through 9, indicating a bimodal distribution. Any other distribution patterns not meeting either threshold were categorized as equivocal.

For statements achieving consensus, the median score was used to determine the level of agreement. Statements with a median score between 7 and 9, with consensus, were classified as agreed on, whereas statements with a median score between 1 and 3, with consensus, were classified as disagreed on. Statements with a median score of 4 through 6, even if consensus was achieved, or any median with nonconsensus, were categorized as uncertain.

For example, if a statement received scores of 7, 8, or 9 from 85% of participants and the remaining 15% scored within 4 through 6, this was classified as consensus agreement (median in 7–9, < 20% outside range). In contrast, if 40% scored 1 through 3 and 60% scored 7 through 9, with a median of 7, this bimodal distribution was classified as nonconsensus. This approach ensured that only statements with clear consensus were considered for agreement or disagreement in the final categorization. Anonymized group feedback, including median scores and representative comments, was shared with participants to allow for reflection and refinement of their responses in subsequent evaluations.

### Supplemental Round

A supplemental round was conducted to address unresolved ambiguities in 2 statements from Delphi round 2. These statements concerned the evaluation of specific AMD lesions using color fundus photography and OCT. Specifically, the question sought to address multimodal assessment of AMD in situations where a lesion is visible on one imaging method, but not the other. In this round, participants re-evaluated these statements with additional clarifying information provided. Responses again were analyzed using the same statistical criteria applied in the earlier round.

## Results

### Delphi Round 1: Identification of Key Issues

The first Delphi round involved qualitative exploration to identify key issues and to gather expert perspectives on reference standards for AMD screening. Responses revealed several areas of agreement and divergence:

- The Beckman classification and AREDS Simplified Severity Scale were acknowledged widely as the most relevant grading systems for AMD, but experts noted the need for an updated classification to incorporate features detectable through multimodal imaging, particularly OCT.
- Presence of reticular pseudodrusen was identified as a feature warranting further discussion, with varying opinions on its potential inclusion in a reference standard.
- The suitability of fundus autofluorescence as an imaging method was debated, with many experts questioning its additional value over OCT and color fundus photography.
- Experts emphasized the importance of distinguishing vision-threatening AMD (e.g., neovascular AMD and central GA) from non–vision-threatening AMD in screening and referral pathways.
- Performance goals of screening devices and dependence of performance metrics on the referral stage were discussed.

These findings informed the development of structured statements for quantitative evaluation in the subsequent round.

### Delphi Round 2 and Supplemental Round: Quantitative Consensus

**Figure 1.**
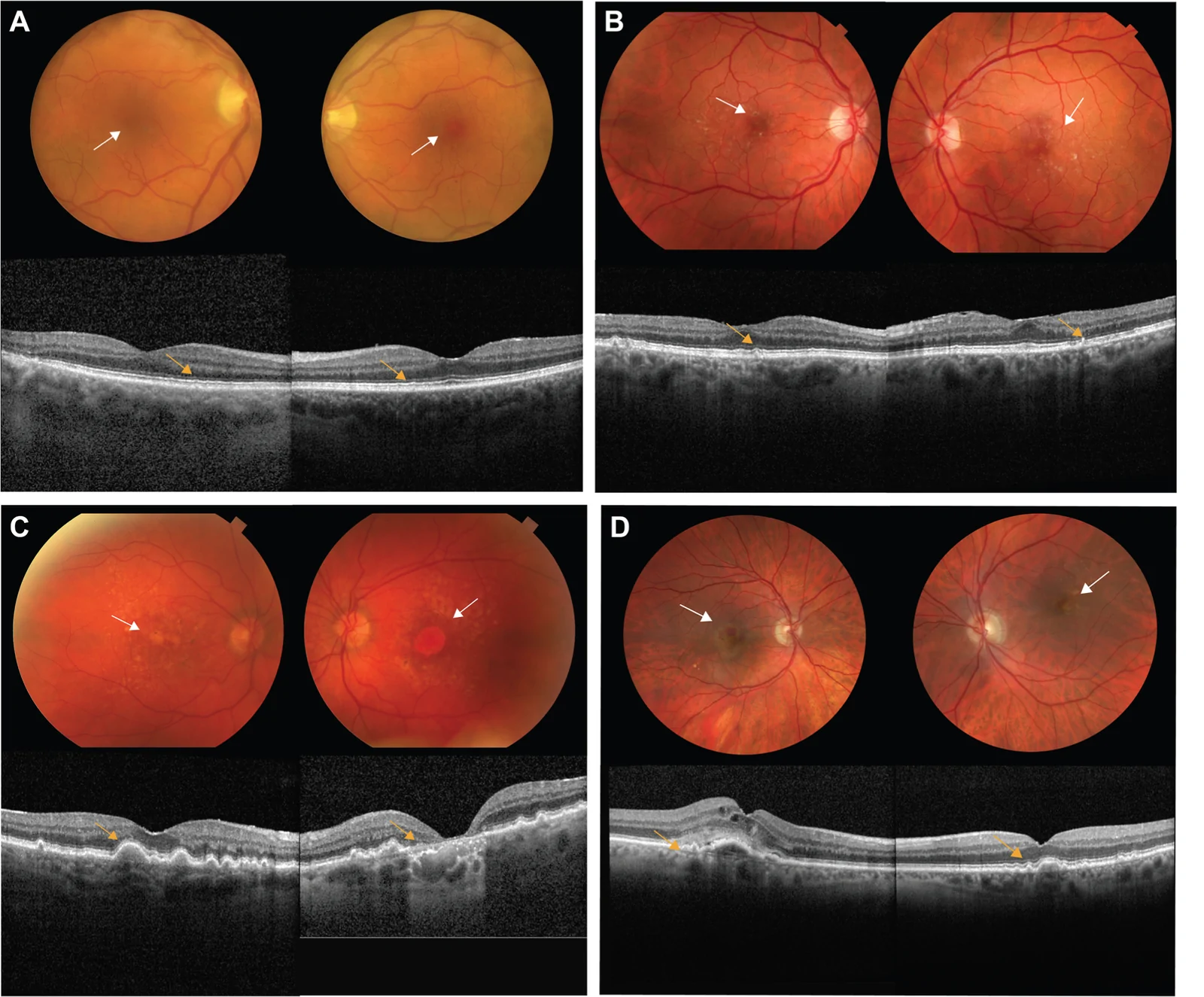
Examples of age-related macular degeneration (AMD) stages on the Beckman scale shown with color photographs and OCT scans: Arrows pointing to AMD lesions in each set of images. Small drusen in both eyes without pigment changes (early AMD; nonreferable) (**A**), medium drusen with pigment changes in both eyes (intermediate AMD; referable, nonurgent) (**B**), large drusen with pigment changes in the right eye (intermediate AMD) and central geographic atrophy in the left eye (late AMD; referable, nonurgent) (**C**), and neovascular AMD in the right eye and large drusen in the left eye (late AMD; referable, urgent) (**D**).

**Figure 2.**
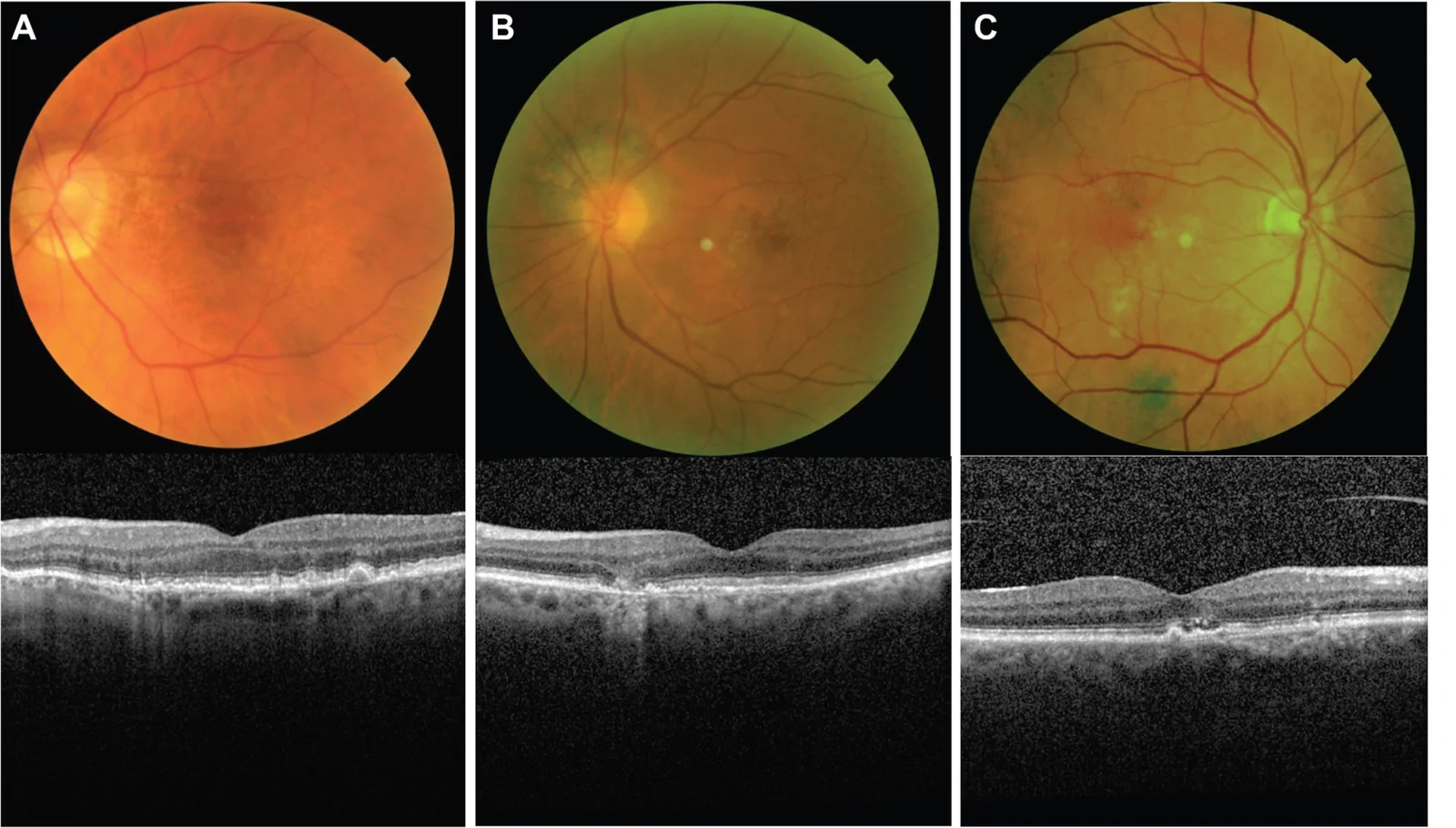
Examples of disagreement in age-related macular degeneration (AMD) features between color photographs and OCT, illustrating the use of a multimodal scale: small drusen on color photograph with large drusen on OCT (intermediate AMD; referable, nonurgent) (**A**), large drusen on color photograph with complete retinal pigment epithelium and outer retinal atrophy (cRORA, geographic atrophy) on OCT (advanced AMD; referable, nonurgent) (**B**), borderline-quality color photograph with large drusen and macular neovascularization with subretinal fluid on OCT (advanced AMD; referable, urgent) (**C**).

The second round focused on achieving consensus on specific statements derived from round 1. A total of 11 statements were analyzed, and consensus was defined using preestablished statistical criteria. Consensus was achieved for most statements, including adoption of the Beckman classification as the reference standard, role of reticular pseduodrusen (RPD) in referrals, incorporation of OCT with color fundus photography, and referral stratification for neovascular versus nonneovascular AMD. Nonconsensus was observed for the minimum age threshold for screening. Summary statistics, including medians, ranges, and score distributions, are shown in Table 1. The supplemental round included questions related to drusen, pigmentary abnormalities, GA, and neovascular AMD in a multimodal evaluation; consensus was achieved for all features except pigmentary abnormalities. Figure 1 and Figure 2 depict examples of multimodal feature assessment and referral status.

## Discussion

The development of a widely accepted reference standards is critical for advancing AI-based tools for AMD screening.^21^ Reference standards not only provide a benchmark for evaluating algorithm performance, but also ensure reproducibility and comparability across studies.^11^ In AMD, in which classification involves nuanced staging, standardization allows for improved evaluation of AI performance. The Delphi process used in this study successfully established consensus on essential elements of an AMD reference standard, including imaging methods, disease staging, and referral pathways, laying the groundwork for future clinical applications and regulatory approval processes.

The Beckman classification emerged as the preferred level 1 reference standard for AMD screening because of its ability to delineate disease stages clearly with high clinical relevance. Importantly, the Beckman categories align closely with the International Classification of Diseases diagnostic structure used in routine clinical care (early, intermediate, GA, and neovascular AMD), allowing it to function as a simplified, image-based labeling framework for screening validation.^22^ Intermediate AMD as defined by the presence of large drusen (> 125 μm) or pigmentary abnormalities associated with medium drusen (> 65–≤ 125 μm) represents a critical point for clinical decision-making.^23^ Intermediate AMD has been shown to carry a significantly higher risk of progression to late stages. Identifying this stage facilitates screening of appropriate patient referral and potential enrollment in interventional clinical trials targeting early disease stages.^24^ Although the field of AMD is evolving rapidly, with multiple new OCT biomarkers potentially related to risk of progression, longitudinal studies and natural history studies with these biomarkers are still evolving.^25,26^ In addition, the Beckman classification is predictive of progression to late AMD and associated visual outcomes, rather than being based solely on reader agreement, allowing meaningful assessment of AI performance.^15^ Currently no strong evidence exists to support use of a classification based solely on OCT. However, consensus emerged on incorporating volumetric OCT in identifying AMD features contributing to the Beckman scale. Although the reference standard incorporates OCT features to improve labeling accuracy, this recommendation does not imply that OCT must be used at the point of screening.

Consensus was reached that drusen detected on OCT, even if not visible on color imaging, should be recorded as present to capture this disease feature. However, a notable challenge identified during the Delphi process was the variability in drusen size measurements between color fundus photography and OCT. While drusen size of more than 125 μm is a critical threshold for classifying intermediate AMD on color imaging, discrepancies may arise when translating this measurement to OCT because of differences in visualization and quantification.^27,28^ In OCT, the cross-sectional scan may not pass through the drusen’s widest diameter, leading to variability in size estimation. When drusen are visible on both methods, consensus was that color fundus photography should serve as the reference for size classification and also that a size of more than 125 μm on OCT imaging is required for classification to avoid overcalling large drusen based solely on OCT.

Other features evaluated with more than 1 method for the Beckman scale include pigment change, GA, and neovascular AMD. Although strong consensus emerged that OCT detects late AMD features with higher sensitivity, the panel was divided on using OCT alone for pigment change. Hyperreflective foci on OCT mark displaced retinal pigment epithelium and have histologic confirmation as intraretinal retinal pigment epithelium migration, yet coregistration studies show strong but incomplete agreement between color hyperpigmentation and hyperreflective foci.^29–31^ Given this lack of one-to-one correspondence, some experts favored retaining color photography as the primary source for pigment assessment, whereas others supported OCT-based detection for clinical precision. Therefore, the panel did not support treating OCT as a standalone surrogate for pigment change within the Beckman scale. For AI developers, OCT-based hyperreflective foci may be incorporated as model inputs, but performance should be benchmarked against color-defined pigment labels to maintain consistency with the current reference standard.

Effective referral pathways are essential for optimizing patient outcomes and ensuring timely intervention in AMD. This study achieved consensus on the need to distinguish between vision-threatening and non–vision-threatening AMD for referral purposes. Vision-threatening AMD includes both neovascular AMD and all GA. However, consensus was that immediate referral, defined as a referral within 1 week, is needed for neovascular AMD. Consensus was that GA, with or without vision loss, should lead to nonurgent referral, such that individuals might benefit from potential treatment, education, monitoring, or lifestyle modifications.^6^ This stratified approach aligns with clinical priorities and ensures that patients receive care appropriate to their disease stage. Urgent referral for suspected neovascular AMD is conceptually similar to the approach used in DR screening, where vision-threatening DR is flagged for prioritized referral. In both cases, the screening output is intended to indicate relative urgency for specialist evaluation, rather than to prescribe fixed timelines for care. During the Delphi process, no consensus emerged about the recommended minimum age for screening, although most favored 50 years or older. This nonconsensus reflected competing priorities; some participants favored earlier screening to identify individuals at risk, whereas others were concerned about increase in screening burden without clear evidence of benefit. The American Academy of Ophthalmology identifies 50 years or older as the typical age of AMD,^6^ whereas the United States Centers for Disease Control and Prevention’s Vision and Eye Health Surveillance System uses 40 years of age or older as a cutoff to estimate AMD prevalence.^1^ The United States Preventive Services Task Force does not support screening for impaired visual acuity among older adults, defined as older than 65 years.^32^ However, they acknowledged that studies to include in their systematic review were limited and that the data relied heavily on the AREDS, because other studies had methodologic limitations. For AI development, diverse age groups should be included during model training and testing, because screening age thresholds will vary across health care systems and countries.

This study’s strengths include the use of a widely accepted Delphi process to achieve consensus among a diverse group of AMD experts. The iterative rounds allowed for refinement of ambiguous points and provided a comprehensive framework for AMD screening reference standards. However, the study has limitations, including the relatively small size of the expert panel and the potential for regional variability in clinical practices. Additionally, some areas, such as the inclusion of pigmentary abnormalities detected solely on OCT, require further investigation to resolve remaining uncertainties.

This study highlights the importance of establishing reference standards for AI-based AMD screening. Similar to DR, where the Early Treatment Diabetic Retinopathy Study grading system and Diabetic Retinopathy Clinical Research Network reference standards enabled regulatory approval of screening tools, adopting a structured AMD reference standard supports FDA expectations around transparency, repeatability, and human-graded comparators. This approach aligns with FDA-endorsed Good Machine Learning Practice principles, which emphasize the use of fit-for-purpose reference standards selected through broad expert consensus and appropriate domain expertise.^33^ Although this consensus does not represent FDA guidance, it provides a framework that can inform regulatory evaluation of future AMD AI submissions. The consensus achieved on imaging protocols, disease classification, and referral pathways provides a framework for validating AI models. Notably, the panel reached agreement on several elements that have not been defined previously for AMD screening. First, consensus support for a multimodal (color photography plus OCT) assessment within a Beckman classification system reflects an important evolution from prior color photography-only approaches and outlines how OCT-derived findings, such as drusen, complete retinal pigment epithelium and outer retinal atrophy (cRORA), and neovascular AMD presence, can be incorporated into AI model training. Second, isolated reticular pseudodrusen without large drusen were not classified as a referable category, providing clearer label boundaries for training models. By integrating these standards into AI validation processes, it should be possible for AMD screening algorithms to identify individuals with disease that is at risk of progressing to or who are at risk of late AMD, to optimize referral systems, and to facilitate timely interventions that preserve vision and quality of life.

A limitation of the proposed reference standard is its foundation on the Beckman classification, which was derived primarily from natural history studies in predominantly White populations in AREDS studies.^15^ Because AMD phenotypes and progression patterns differ across Asian and other cohorts, additional data and validation work will be required to determine whether the same scales, multimodal imaging thresholds, and referral cutpoints are generalizable globally. Further work is required to test the updated Beckman scale with multimodal imaging in diverse and primary care screening settings and the role of emerging biomarkers in establishing a new classification of AMD. Developing AI algorithms aligned with the current consensus-based reference standards will enhance the accuracy and scalability of AMD screening, ultimately reducing the burden of late-stage disease.

## Supplementary Material

MMC2

MMC1

MMC3

Supplemental material available at www.aaojournal.org.

## Figures and Tables

**Table 1.** Median and Agreement Status for Each Statement

No.	Statement	Median	Range	Consensus	Final Classification

1	The Beckman scale is appropriate as a reference standard for validating AI algorithms used in population screening of AMD.	8	Range, 5—9 7—9, 9 (82%) 4—6, 2 (18%) 1—3, 0	Consensus	Agreement
2	The Beckman scale assessment is primarily conducted using color photographs and needs to be updated to incorporate multimodal assessment with volumetric OCT for certain features.	9	Range, 8—9 7—9, 11 (100%) 4—6, 0 1—3, 0	Consensus	Agreement
3	Fundus autofluorescence imaging does not add additional value to the assessment of the Beckman scale in addition to color photography and OCT.	8	Range, 4—9 7—9, 7 (64%) 4—6, 4 (36%) 1—3, 0	Consensus	Agreement
4	There is no prognostic relevance for incorporating reticular psedudrusen in the reference standard assessment.	7	Range, 2—9 7—9, 8 (73%) 4—6, 1 (9%) 1—3, 2 (18%)	Consensus	Agreement
5*	Drusen not visible on color photographs but detected on OCT should be recorded as present.	8	Range, 1—9 7—9, 5 (62%) 4—6, 1 (12%) 1—3, 2 (25%)	Consensus	Agreement
6*	Pigment changes not visible on color photographs but detected on OCT should be recorded as present.	7.5	Range, 1—9 7—9, 4 (50%) 4—6, 2 (25%) 1—3, 2 (25%)	Nonconsensus because of bipolar distribution	Uncertain
7*	Geographic atrophy not visible on color photographs but detected as complete retinal pigment epithelium and outer retinal atrophy (cRORA) on OCT should be recorded as present.	8.5	Range, 3—9 7—9, 7 (88%) 4—6, 0 1—3, 1 (12%)	Consensus	Agreement
8*	Neovascular AMD not visible on color photographs but detected on OCT should be recorded as present.	9	Range, 7—9 7—9, 8 (100%) 4—6, 0 1—3, 0	Consensus	Agreement
9	AI-based screening for AMD should be deployed in individuals older than 50 years.	8	Range, 3—9 7—9, 8 (73%) 4—6, 2 (18%) 1—3, 1 (9%)	Nonconsensus because of bipolar distribution	Uncertain
10	For AI-based population referral, individuals with intermediate AMD or late AMD should be referred for further evaluation.	9	Range, 3—9 7—9, 10 (91%) 4—6, 0 1—3, 1 (9%)	Consensus	Agreement
11	An effective referral system should distinguish GA and CNV for the category of late AMD and urgency of referral should be categorized as no referral, referral needed for intermediate AMD and GA, and immediate referral needed for CNV.	9	Range, 7—9 7—9, 11 (100%) 4—6, 0 1—3, 0	Consensus	Agreement

AI = artificial intelligence; AMD = age-related macular degeneration; CNV = choroidal neovascularization; GA = geographic atrophy.

* Part of supplemental round and had 8 participants.

## Abbreviations and Acronyms:

AI: artificial intelligence
AMD: age-related macular degeneration
AREDS: Age-Related Eye Disease Study
DR: diabetic retinopathy
FDA: Food and Drug Administration
GA: geographic atrophy
