# Supplementary material for: Reference Standard for Validation of Age-Related Macular Degeneration Screening Algorithms
Source: Ophthalmology. Author manuscript; Available in PMC 2026 Aug 22. (PMC13498439; doi:10.1016/j.ophtha.2026.04.013)
Supplement: Supplementary file 1 [file NIHMS2167038-supplement-MMC1.pdf]

Supplement 1: Qualitative Exploration

The following topics and open-ended discussion prompts were shared with expert panelists during the preparatory phase before initiating the formal Delphi process. These materials were designed to align understanding, surface known definitions and frameworks, and ensure familiarity with key issues in AMD screening. The discussion was staged around the following topics.

Theme	Summary of discussion
1. Clinical Reference Standards: Are current classification systems (e.g., AREDS detailed scale, AREDS simplified scale, Beckman scale, AAO AMD definition) appropriate for defining a clinical reference standard for AMD?	Round 1 feedback acknowledged the value of existing AMD scales (AREDS detailed scale, AREDS simplified, Beckman), but opinions varied on which framework was most suitable for screening validation. The AREDS detailed scale was not favored by many because of its complexity . Since reticular pseudodrusen were not part of previous scale, it emerged as a topic of importance.
2. Imaging Modalities: Should newer imaging modalities and multimodal assessment be incorporated in addition to color photography? What is the role of OCT and Fundus autofluorescence in AMD screening? What imaging protocols are suitable for AMD screening?	Participants discussed expanding beyond color fundus photography to include multimodal imaging, particularly OCT, given its widespread clinical use and ability to detect neovascular AMD. They noted that existing AMD scales are primarily color based, and the absence of OCT-based grading frameworks remains a challenge for multimodal reference standard development. This feedback informed Round 2 statements focused on the relative roles of color photography and OCT, and the need to specify lesion level definitions across modalities.
3. Target Screening Population: What patient population should AMD screening devices target?	Discussion was focused on appropriate age to balance detection yield and resource burden. Country policies in various parts of the world were also discussed.
4. Referral Classification and timeframes: Who should be	Experts debated how AMD should be staged for referral decisions, including

referred? Should AMD be staged to identify vision-threatening diseases, similar to diabetic retinopathy? Should neovascular AMD and geographic atrophy be separated in screening.	whether neovascular AMD and geographic atrophy should be separated, and whether vision-threatening disease should be prioritized. There was discussion around a binary referral, a 3 level and 5 level referral system.
5. Performance metrics overall and by disease stage: What performance thresholds should AMD screening devices meet? Should performance thresholds differ by AMD severity (e.g., early vs. vision-threatening)?	Panelists generally agreed that accuracy thresholds for screening devices should differ by disease stage, and higher accuracy for vision-threatening AMD.
