# Supplementary material for: Reference Standard for Validation of Age-Related Macular Degeneration Screening Algorithms
Source: Ophthalmology. Author manuscript; Available in PMC 2026 Aug 22. (PMC13498439; doi:10.1016/j.ophtha.2026.04.013)
Supplement: Supplementary file 2 [file NIHMS2167038-supplement-MMC3.pdf]

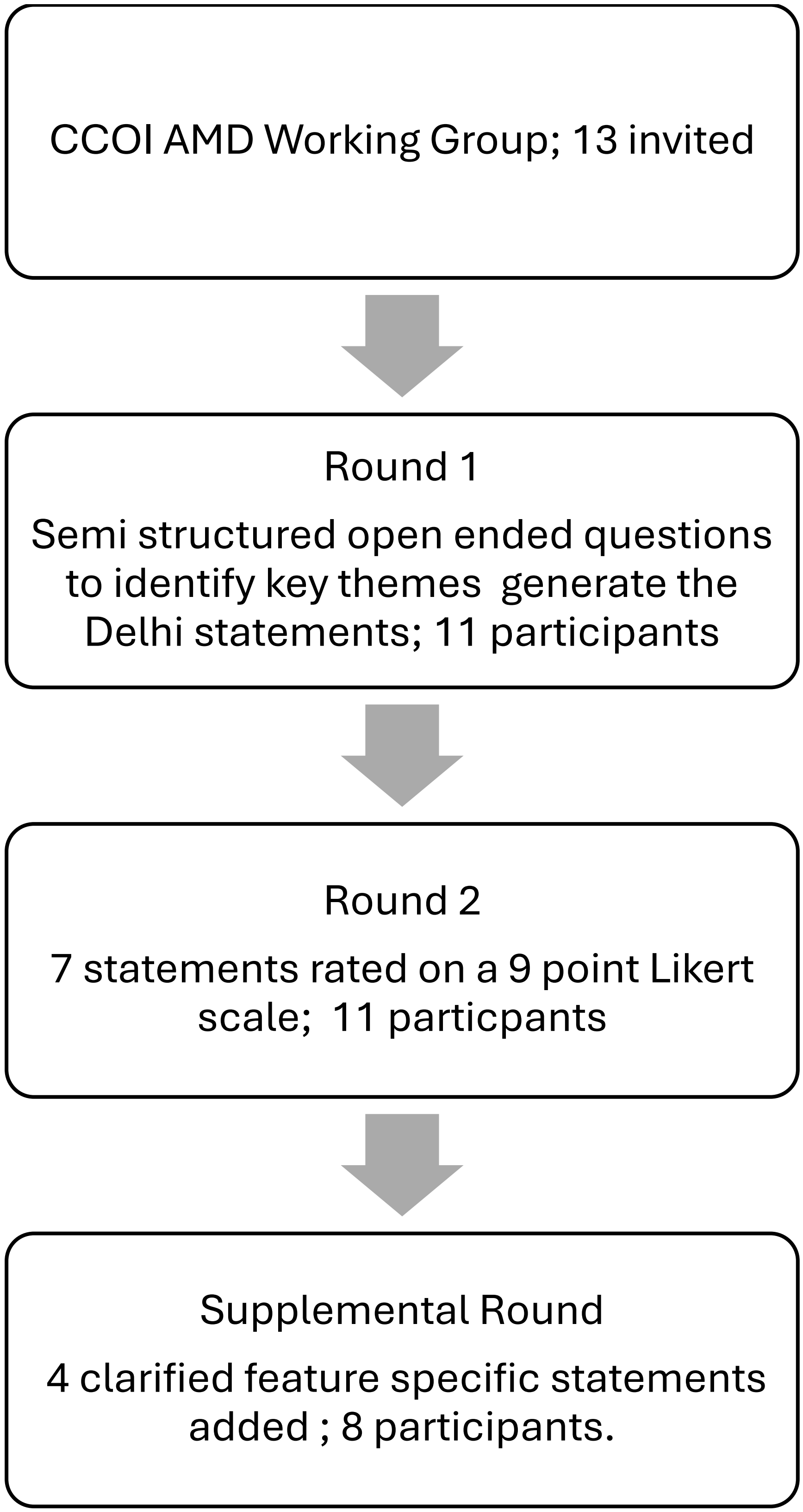
CCOI AMD Working Group; 13 invited
Round 1
Semi structured open ended questions to identify key themes generate the Delhi statements; 11 participants
Round 2
7 statements rated on a 9 point Likert scale; 11 particpants
Supplemental Round
4 clarified feature specific statements added ; 8 participants.
