# Supplementary material for: Reference Standard for Validation of Age-Related Macular Degeneration Screening Algorithms
Source: Ophthalmology. Author manuscript; Available in PMC 2026 Aug 22. (PMC13498439; doi:10.1016/j.ophtha.2026.04.013)
Supplement: Supplementary file 3 [file NIHMS2167038-supplement-MMC2.docx]

**Appendix 1**

**Working group for Collaborative Community for artificial intelligence (CCOI_ in age-related macular degeneration:**

Emily Chew, MD (Chair, National Eye Institute/National Institutes of Health)

Bailey Freund, MD

Mingguang He, MD, PhD

Pearce Keane, MD

Tiarnan Keenan, BM BCh, PhD

Eleonora Lad, MD, PhD

Aaron Lee, MD

Cecilia Lee, MD

Jennifer Lim, MD

Anat Loewenstein, MD

Theodore Smith, MD

**CCOI Executive Committee:**

Michael Abramoff, MD, PhD

Mark Blumenkranz, MD

Emily Chew, MD

Michael Chiang, MD

Malvina Eydelman, MD

Aaron Lee, MD

David Myung, MD, PhD

Michael Repka, MD

Joel S. Schuman, MD

Carol Shields, MD

CCOI working group
